# Thioredoxin-dependent regulatory networks in chloroplasts under fluctuating light conditions

**DOI:** 10.1098/rstb.2013.0224

**Published:** 2014-04-19

**Authors:** Lauri Nikkanen, Eevi Rintamäki

**Affiliations:** 1 Molecular Plant Biology, Department of Biochemistry, University of Turku, Turku 20014, Finland; 2 Department of Biological and Environmental Sciences, University of Gothenburg, Gothenburg, Sweden

**Keywords:** chloroplast, thioredoxin, redox network, fluctuating light, reactive oxygen species, environmental signals

## Abstract

Plants have adopted a number of mechanisms to restore redox homeostasis in the chloroplast under fluctuating light conditions in nature. Chloroplast thioredoxin systems are crucial components of this redox network, mediating environmental signals to chloroplast proteins. In the reduced state, thioredoxins control the structure and function of proteins by reducing disulfide bridges in the redox active site of a protein. Subsequently, an oxidized thioredoxin is reduced by a thioredoxin reductase, the two enzymes together forming a thioredoxin system. Plant chloroplasts have versatile thioredoxin systems, including two reductases dependent on ferredoxin and NADPH as reducing power, respectively, several types of thioredoxins, and the system to deliver thiol redox signals to the thylakoid membrane and lumen. Light controls the activity of chloroplast thioredoxin systems in two ways. First, light reactions activate the thioredoxin systems via donation of electrons to oxidized ferredoxin and NADP^+^, and second, light induces production of reactive oxygen species in chloroplasts which deactivate the components of the thiol redox network. The diversity and partial redundancy of chloroplast thioredoxin systems enable chloroplast metabolism to rapidly respond to ever-changing environmental conditions and to raise plant fitness in natural growth conditions.

## 1. Introduction

**Figure 1.**
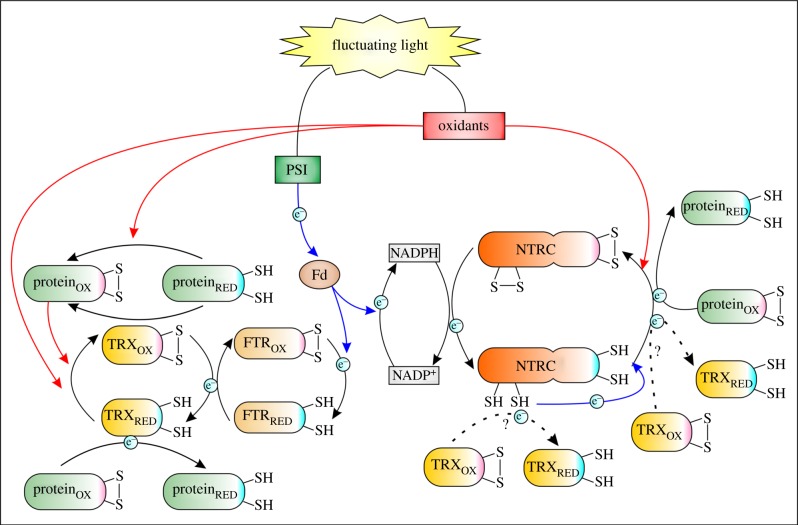
Redox network of chloroplast thioredoxin systems in fluctuating light. Light controls the activity state of thioredoxin systems via reduction of ferredoxin (Fd) and via production of oxidants in chloroplasts. Chloroplast proteins are reduced either by ferredoxin-dependent (FTR + TRX) or NADPH-dependent (NTRC) thioredoxin system. The thioredoxin reductase domain of NTRC reduces the thioredoxin domain, or hypothetically, donates electrons to free TRXs. Oxidants control thiol redox homeostasis by oxidizing dithiols both in proteins and in TRXs. Reducing signals are depicted as blue arrows, oxidizing signals as red arrows and hypothetical electron transfers as dashed arrows.

Covalent modification of the amino acid side chain is the most common post-transcriptional mechanism to control protein function in cells. Formation and cleavage of a disulfide bond between the side chains of two cysteine residues (Cys) regulate both the structure and function of cellular protein. The Cys content in proteins has increased during their evolution, and the Cys forming disulfide bonds are more conserved than those not involved in redox regulation [1,2]. Thioredoxins (TRXs) are protein oxidoreductases that have a redox active dithiol/disulfide motif in their active site. In the reduced state, they induce reductive cleavage of a disulfide bond in target proteins via a bimolecular nucleophilic substitution reaction. TRXs that become oxidized in the reaction are reduced by thioredoxin reductases (TRs; figure 1). TR-dependent reduction of TRXs is called a TRX system. Plants are distinguished from other organisms by having highly versatile TRX systems, including two TRs dependent on ferredoxin (FTR) and NADPH (NTR) as reducing power, respectively, and multiple types of TRXs (h, o, f, m, x, y, z, and several TRX-like proteins) [3,4]. The majority of these TRXs are localized to plant chloroplasts, which emphasizes the impact of TRX-dependent regulation of proteins in chloroplasts. More than 20 genes encoding chloroplast TRXs have been identified in *Arabidopsis* and poplar genomes [4,5]. Evolution of diverse TRX systems is likely linked to high redox activity and oxygenic metabolism in chloroplasts [1]. A number of comprehensive reviews on chloroplast TRXs have lately been published [4–9]. In this review, we address the impact of chloroplast TRX systems on the redox signalling in fluctuating light conditions with specific focus on thiol-redox networks from stroma to thylakoids and lumen.

## 2. Chloroplast thioredoxin systems

**Figure 2.**
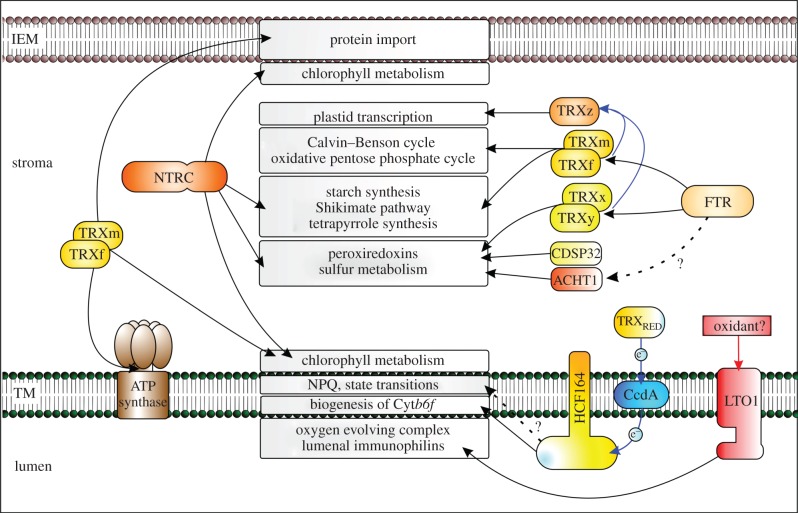
Chloroplast processes controlled by TRX systems. Experimentally established processes in stroma, thylakoid membranes (TM), lumen and in inner envelope membrane (IEM) as well as the TRXs mediating the regulation are included in the figure. The enzymes catalysing chlorophyll metabolism are associated both with thylakoid and inner envelope membranes. CcdA and HCF164 proteins transmit the reducing signal from stroma to lumen. For details, see §2.

Five types of classical low-molecular weight TRXs (f, m, y, x, z; figure 2) have been reported for chloroplasts, of which f, m and y exist in *Arabidopsis* as two, four and two isoforms, respectively [3,10]. TRX f is of eukaryotic and m, y, x and z of prokaryotic origin, whereas the redox active sequence in all classical TRXs is WCGPC [1,10,11]. The chloroplast also contains TRX-like proteins with longer amino acid sequence and modifications in the redox active site sequence [4,5,11]. CDSP32 is a TRX-like, drought-induced stress protein localized to chloroplast stroma [12]. Five *Arabidopsis* genes encode Lilium-type TRX-like proteins [4] that have recently been named as AtACHTs (atypical Cys His-rich TRXs) [11,13], and two TRX-like proteins called WCRKC have been reported to exist in chloroplast stroma [4,13]. Prokaryotic HCF164 was the first TRX-like protein localized to thylakoids [14]. The redox active site of HCF164 is on the lumenal side of the thylakoid membrane and it has been proposed to transmit redox signals from stroma to lumenal proteins together with the thylakoid protein CcdA [15,16]. CcdA has no TRX motif, but it is homologous to bacterial proteins that assist disulfide formation in secreted proteins by mediating movement of redox equivalents between bacterial cytoplasm and periplasm [15]. Recently, a novel TRX-like thylakoid protein has been reported. Thylakoid-localized lumen thiol oxidoreductase 1 (LTO1) consists of two domains, from which the N-terminal domain is homologous to mammalian vitamin K reductase [17,18]. The second domain has a TRX-like sequence with homology to bacterial thiol oxidoreductase protein DsbA [18] which is a member of the same prokaryotic thiol disulfide transporter system as CcdA [19].

Two thioredoxin reductases exist in chloroplasts, using ferredoxin (FTR) or NADPH (NTRC) as an electron donor in the reduction of the TRX active site (figure 1) [6,20]. FTR is an iron–sulfur protein with a redox active disulfide bridge capable of mediating electron transfer from ferredoxin to the disulfide bridge in TRXs [1,21]. Chloroplast NADPH-dependent NTRC enzyme is unique for oxygenic photosynthetic organisms. It forms a complete TRX system with a TRX sequence fused to the C-terminus of an NTR sequence (figure 1) [20]. It has been reported that NTRC mainly reduces its own TRX with inefficient interaction with free chloroplast TRXs [22,23], suggesting that FTR is the main reductant for free chloroplast TRXs. Recently, however, based on experiments with transgenic *Arabidopsis* lines overexpressing *NTRC* with an inactive TRX domain, it was suggested that NTRC can donate electrons to free TRXs [24]. Reduction of TRX f, m, y and x by FTR has been documented, whereas neither FTR nor NTRC can reduce TRX z (figure 2) [6,23]. TRX z has a unique surface structure that may explain its inability to interact with TRs [24]. TRX z activity probably relies on the other free chloroplast TRXs (figure 2) [23]. TRX f and m can also donate electrons to CcdA [16], whereas the reduction system for the other thylakoid TRX-like proteins in thylakoid membranes is not yet known. Finally, reduction of AtACHT1 is light-dependent, but the primary electron donor for AtACHT1 or other AtACHTs is not yet known [25].

## 3. Targets of chloroplast thioredoxin systems

The ferredoxin–thioredoxin system with TRX f and m was originally demonstrated to control carbon metabolism in dark/light transition by activating distinct enzymes in the Calvin–Benson cycle and inactivating glucose 6-phosphate dehydrogenase in an oxidative pentose phosphate pathway [6]. Later, TRX-regulated enzymes were found in other metabolic pathways, including starch metabolism, chloroplast energetics, nitrogen and sulfur metabolism, as well as Shikimate and tetrapyrrole pathways (figure 2). TRX x, y and CDSP32 are assigned to control stress responses in chloroplasts. They are all involved in antioxidant metabolism by reducing oxidized 2-Cys peroxiredoxins, whereas CDSP32 and TRX y also donate electrons to enzymes in sulfur metabolism [26,27] (figure 2). TRX z controls plastid transcription by regulating plastid-encoded RNA polymerase via an unknown mechanism [10]. Proteomic approaches using TRX baits (for methods, see the recent review by Lindahl *et al*. [28]) have revealed over 400 potential TRX target proteins in plant cells, of which around 100 are localized to chloroplasts [6,8,29]. These targets cover all major functions of the chloroplast from biogenesis, DNA metabolism, translation and protein folding to secondary metabolic pathways. Redox regulation of these target proteins still needs experimental verification, because proteomic techniques are prone to false-positive signals.

The NTRC system is a crucial thiol redox regulator both in chloroplast and non-photosynthetic plastids [22,30,31]. The severe phenotype of knockout lines of *NTRC* demonstrated that the NTRC system is non-redundant to FTR in chloroplasts [22,30]. The H_2_O_2_-detoxification enzymes 2-Cys-peroxiredoxins and ADP-glucose pyrophosphorylase, which catalyses the key reaction in starch synthesis, are the most conclusively documented target proteins of NTRC [22,32,33]. Characterization of the knockout line of *NTRC* has revealed other plastidial processes as potential targets of NTRC regulation, including biogenesis of chloroplasts and biosynthesis of tetrapyrrole and aromatic amino acids (figure 2) [30,34,35].

Besides stromal pathways, processes subjected to TRX regulation have been identified both in inner envelope, thylakoid membranes and lumen (figure 2). Redox control of the components in the protein import machinery localized to inner envelope membrane (Tic110, Tic55, PTC52) has been recently reported [36,37]. The enzymes involved in chlorophyll biosynthesis [35,38] and degradation [36] localized both to inner envelope and thylakoid membranes are regulated by TRX systems (figure 2). Chloroplast TRXs also control the formation of the cytochrome *b6f* (Cyt*b6f*) complex via the redox-regulation of cytochrome *f* maturation [15], state-transition-associated LHCII protein phosphorylation [39,40], and ATP synthesis via modification of the γ-subunit of ATP synthase [41]. Of the lumenal targets, TRX-dependent regulation of violaxanthin de-epoxidase (VDE) in the xanthophyll cycle [42] and immunophilins [43] that assist protein folding have been experimentally verified. TRX target proteins in lumen and the lumenal side of thylakoids are likely regulated by membrane-bound TRXs, because no TRXs have been found in lumen. HCF164 and LTO1 are potential candidates to transmit redox signals from stroma to lumen, because the domains containing redox-active cysteines are located on the lumenal side of the thylakoid membranes (figure 2) [16,17].

## 4. Chloroplast thioredoxin systems in fluctuating light

Chloroplast TRX systems form an elegant mechanism to couple light and carbon fixation reactions in order to balance the energy conversion and consumption reactions in photosynthesis [3]. Much less attention has been paid to the regulation of chloroplast enzymes under fluctuating light intensity or to the dark-induced deactivation mechanisms of the enzymes. This is largely due to technical difficulties in accurately determining the redox state of TRXs and their target proteins *in vivo*. However, several facts speak for alteration of redox state of TRX target proteins under fluctuating redox conditions in the chloroplast. Both molecular oxygen and oxidized TRXs have been suggested to cause deactivation of redox-controlled chloroplast enzymes [6]. Molecular oxygen has been shown to speed up the deactivation of fructose 1,6-bisposphatase fourfold in comparison with anaerobic conditions [6]. Thereby, the enzymes activated by TRXs are not ‘permanently’ active in light, but need a continuous supply of electrons from TRX systems to maintain the active form (figure 1). If the rate of electron flow from light reactions diminishes, then part of the enzyme pool remains deactivated. Furthermore, accumulation of oxidants, e.g. oxidized 2-Cys peroxiredoxins, has recently been shown to challenge chloroplast TRX systems. Dangoor *et al*. [25] reported that illumination of a leaf induced first the reduction of ACHT1 that was subsequently oxidized by oxidized 2-Cys peroxiredoxins under continuous illumination. The oxidation occurred faster under moderate light intensity. This transient reduction of chloroplast TRX allows light-intensity-dependent regulation of redox active target proteins (figure 1). Fairly similar redox potentials of TRXs and the redox active disulfide of target proteins (see e.g. table 1 in [6]) enable reversibility of the reaction between the TRX and its target proteins. Substrate binding or small changes in stromal pH may also influence the ambient midpoint potential of a redox-regulated enzyme [6], facilitating reversibility of the redox reaction.

Transient changes in the activity of the components involved in state transitions and non-photochemical quenching (NPQ) are illustrative examples of TRX-dependent regulation under fluctuating light intensity. State transitions control the equal distribution of light energy to photosystems I and II under low light, whereas NPQ dissipates excess energy absorbed by the light harvesting complex (LHC) in high light conditions [44,45]. In state transition, reduction of the plastoquinone (PQ) pool (called state 2) by light activates LHCII protein phosphorylation, whereas oxidation of the PQ pool (called state 1) induced dephosphorylation of LHCII antenna proteins. This reversible phosphorylation of LHCII proteins is catalysed by thylakoid-localized STN7 protein kinase and PPH1/TAP38 protein phosphatase [46–48]. According to the classical theory of state transitions, phosphorylation of LHCII proteins protects PSII from over-excitation in high light conditions by inducing transition of phosphorylated LHCII trimers from PSII to PSI and thereby decreasing and increasing the light harvesting capacity in PSII and in PSI, respectively [49]. State transition theory has been revised [44] after discovery of light intensity-dependent inhibition of LHCII protein phosphorylation *in vivo* [50]. The highest accumulation of phosphorylated LHCII protein occurs in leaves illuminated in light intensities lower than growth light, whereas LHCII protein phosphorylation is inhibited after transfer of the plant to higher irradiances than growth light. According to the novel insight of state transitions [44], phosphorylated LHCII proteins harvest light energy for both PSII and PSI that maintains the redox balance in the electron transfer chain under light conditions limiting photosynthesis (low light). In excess light, the mechanism distributing light energy equally to photosystems is futile and therefore must be switched off. STN7 kinase is targeted to two different redox regulations. STN7 kinase is a transmembrane protein that is associated with the Cyt*b6f* complex with a catalytic site on the stromal side and putative redox active cysteines on the lumenal side of thylakoid membranes [51]. Site-directed mutagenesis of the lumenal cysteines totally abolished the kinase activity of STN7 [51,52], indicating that they are essential for the catalytic reaction. The enzyme is activated in light by binding of reduced PQ to the lumenal Qo site in Cyt*b6f* [49,51]. In high light conditions, phosphorylation of LHCII proteins is switched off by TRX-dependent deactivation of STN7 kinase [39,53]. TRX treatment inhibits LHCII protein phosphorylation in isolated thylakoid membranes, whereas hydrogen peroxide recovers the activity [39,53], indicating that STN7 activity is coupled to thiol redox homeostasis in chloroplasts.

If the rate of light absorption by pigments in thylakoid membranes exceeds that used in chloroplasts, then NPQ is activated, which dissipate the excess energy as heat. In plants, NPQ consists of three major components, energy-dependent (qE), zeaxanthin-dependent (qZ) and photoinhibitory (qI) quenching (see [45] for a recent review). Zeaxanthin is involved both in the induction of qE and qZ. Zeaxanthin belongs to the xanthophyll pigments that are bound to chlorophyll *a*/*b* binding proteins in the PSII antenna. Zeaxanthin has been proposed to contribute to NPQ induction by two mechanisms, indirectly by modulating protein/protein interactions in the LHCII complex and directly by quenching excited chlorophylls. High light illumination induces rapid formation of zeaxanthin via de-epoxidation of violaxanthin, another xanthophyll pigment that is saturated within 30 min under moderate and high light illumination [45]. The reaction is catalysed by VDE localized to lumen. VDE is activated by acidification of lumen in light that induces binding of the enzyme to the thylakoid membrane. Reduction of the Cys-rich N-terminal domain of the enzyme by TRX deactivates the enzyme [42,54]. After induction the zeaxanthin level stays stable in high light conditions [45], whereas it is not known if VDE is still active (oxidized form) or deactivated (reduced form) under prolonged illumination.

The redox regulation mechanisms of the VDE and the STN7 kinase resemble each other. Both enzymes have been reported to form homodimers [52,55]. Activation of the enzymes is coupled to light-induced electron transfer in thylakoid membranes, whereas TRX-induced reduction of redox active cysteines deactivates the enzyme. In natural fluctuating light conditions, both enzymes likely are transiently active in thylakoid membranes, VDE being activated rapidly in the induction of NPQ after transfer to high light, whereas STN7 activity responds to changes in light intensity. TRX induces the deactivation of the enzymes by transmitting the redox signal formed in chloroplasts under fluctuating light conditions. STN7 forms intra- and intersubunit disulfides [52], which likely are targeted to TRX-dependent regulation. Reduction of these disulfides may induce monomerization of STN7 or, alternatively, abolish its association with the Cyt*b6f* complex, which deactivates the enzyme. Furthermore, the dithiol form of STN7 may be prone to proteolysis, because loss of STN7 protein in prolonged high light illumination has been reported [56].

Reversible disulfide to dithiol conversion in VDE and STN7 takes place in the thylakoid lumen, where no soluble TRXs have been found. The question is how the redox signal induced by fluctuating light is transmitted to the thylakoid lumen. LHCII protein phosphorylation is inhibited in isolated thylakoids treated with TRX f and m [39], indicating that a thiol redox signal is transmitted through thylakoid membranes from stroma to lumen. A signal transduction pathway using stromal TRXs and transmembrane proteins CcdA and HCF164 is the most probable candidate to transmit reducing equivalents from stroma to redox sites of proteins in the lumen (figure 2). It has been shown that the thiol-alkylating reagent *N*-ethylmaleimide inhibits LHCII protein phosphorylation in thylakoids isolated from high-light-treated leaves, but not in thylakoids isolated from dark-adapted leaves [53], indicating that high light intensity modifies the thiol redox poise of thylakoid membranes.

Another question is, whether the molecular oxygen produced in light reactions is sufficient to reoxidize dithiols in STN7 and VDE or whether they need auxiliary proteins to recover the oxidized form. Knockout lines of thylakoid thiol oxidoreductase LTO1 display a stunted phenotype [17,57], indicating that it has a major impact on chloroplast function. LTO1 is a potential oxidant of dithiols in lumenal proteins (figure 2), because it has a high redox potential [9] and has been reported to assist disulfide formation in lumenal PsbO and immunophilin FKBP13 *in vitro* [17,57].

## 5. Impact of chloroplast thioredoxin systems on plant fitness

Plants have adopted a number of mechanisms that restrain the chloroplast from under- and over-excitation under fluctuating light conditions and rapidly restore redox homeostasis before generation of harmful side products. Chloroplast TRX systems belong to this redox network transmitting environmental signals to chloroplast proteins and thus raise plant fitness in natural growth conditions.

Recent reports focusing on the overexpression of chloroplast TRX components in plants support the concept of a high impact of TRXs on plant fitness. Transformation of tobacco plastid genome with *TRX f* or *TRX m* gene resulted in over 20-fold higher accumulation of TRX f and m proteins in chloroplasts in comparison with wild-type plants [58]. Transgenic tobacco lines overexpressing *TRX f* showed high increase in leaf biomass yield and starch content, which was further stimulated by increase in light intensity, whereas no significant changes were detected in transgenic lines overexpressing *TRX m* [58]. Overexpression of the endogenous *NTRC* gene in *Arabidopsis* nuclear genome also increased growth by maximally doubling leaf biomass yield under moderate light intensity and stimulating starch accumulation in leaves [24]. The extra vitality of the overexpression lines of *TRX f* or *NTRC* was neither assigned to a specific TRX-controlled pathway in chloroplast nor associated with increased steady-state photosynthesis rate per leaf area [58]. Both TRX f and NTRC control several, primarily biosynthetic processes in the chloroplast (figure 2), suggesting that the extra amount of TRX f or NTRC generally boosts chloroplast anabolic metabolism. Alternatively, increased biomass yield in *NTRC* and *TRX f* overexpression lines is due to the chaperone function of TRXs that have recently been assigned to TRX f, m and NTRC [59,60]. However, neither hypothesis explains why overexpression of *TRX m* failed to stimulate growth identically to *NTRC* and *TRX f*. The molecular background for the improved fitness in plants overexpressing TRX f and NTRC remains to be elucidated in the future.

## Funding statement

This work was supported by the Academy of Finland (130192) and by Carl and Thecla Lamberg Foundation.
